# The immediate effects of thoracic spine manipulation in patients with neck pain: a meta-analysis of randomized controlled trials

**DOI:** 10.3389/fmed.2026.1790614

**Published:** 2026-03-31

**Authors:** Yiyun Pan, Jingwen Chen, Yu Liu, Zhengcong Chen, Jie Liu

**Affiliations:** 1 Ganzhou Cancer Hospital, Gannan Medical University, Ganzhou, Jiangxi, China; 2 Ganzhou Hospital-Nanfang Hospital, Southern Medical University, Ganzhou, Jiangxi, China; 3 The First Affiliated Hospital of Gannan Medical University, Ganzhou, Jiangxi, China

**Keywords:** cervical discomfort, immediate effects, meta-analysis, rehabilitation, thoracic manual therapy, thoracic spine manipulation

## Abstract

**Background:**

The immediate therapeutic impact of thoracic spine manipulation (TSM) on pain levels, cervical mobility, and functional impairment in patients with neck pain has not been conclusively established.

**Objective:**

This study aimed to assess the short-term outcomes of TSM by contrasting it with sham interventions or alternative treatment approaches for neck pain management.

**Methods:**

A comprehensive literature review was performed across multiple databases including PubMed, Embase, Web of Science, PEDro, and Cochrane Library, with searches current through January 30, 2023. The analysis focused on randomized controlled trials (RCTs) examining the acute effects of interventions on neck pain patients. Two researchers independently extracted relevant data from selected studies. The primary outcome measure was the difference in clinical measurements taken before and after intervention. Statistical analyses were performed using Review Manager 5.40 software, with the choice of fixed-effect or random-effects model guided by *I*^2^ values.

**Results:**

The study incorporated 17 randomized controlled trials involving 1,100 participants. The meta-analytical results demonstrate that thoracic spine manipulation (TSM) produces notable improvements in pain reduction (standardized mean difference [SMD] = −0.53; 95% confidence interval: −0.84 to −0.22). Significant enhancements were also observed in cervical range of motion across multiple dimensions: forward bending (SMD = 4.27; 95% CI: 2.14–6.40), extension (SMD = 2.33; 95% CI: 0.17–4.49), left side bending (SMD = 2.29; 95% CI: 0.47–4.10), right side bending (SMD = 2.94; 95% CI: 1.09–4.78), left rotation (SMD = 3.15; 95% CI: 0.82–5.47), and right rotation (SMD = 2.47; 95% CI: 0.18–4.76). Additionally, functional disability showed marked improvement (SMD = -7.31; 95% CI: −10.01 to −4.61).

**Conclusion:**

Current evidence, ranging from moderate to strong levels, indicates that TSM yields substantial immediate benefits for individuals experiencing cervical discomfort, including pain relief, improved neck mobility, and reduced functional limitations. The intervention demonstrates excellent safety profiles, making it suitable for widespread clinical application.

## Introduction

The Global Burden of Disease Study reveals that cervical pain occupies the 21st position among 291 medical conditions and stands fourth in terms of disability impact, highlighting its significance as a prevalent health concern requiring immediate attention (1, 2). This condition can be categorized based on temporal characteristics (acute cases lasting under six weeks, subacute persisting up to three months, and chronic exceeding three months) (3). Regardless of duration, cervical discomfort imposes substantial physical and psychological distress on affected individuals, with approximately half the global population reporting persistent or recurrent symptoms (3). Multiple etiological factors contribute to cervical pain manifestations, including improper study habits, ergonomically unsound work positions, and prior cervical injuries (4–7). In addition, long-term pain and dysfunction, can make the patient’s body function decline, causing negative feelings such as depression and anxiety (8). Individuals experiencing cervical discomfort frequently opted for hands-on therapeutic interventions to address their symptoms. Current medical guidelines recommend physiotherapy as the primary intervention for cervical pain management (9). Research has demonstrated the effectiveness of thoracic vertebral manual therapy techniques, especially thrust-based mobilization and manipulation approaches, for cervical pain sufferers. Thoracic spinal manipulation (TSM) represents a hands-on treatment modality employing controlled force and guided motions to address the articular structures and soft tissues of the upper to middle back region (10). In short and long-term treatment of patients with different stages of neck pain, there is substantial evidence that chest thrust manipulation is superior to chest non-thrust mobilization, infrared radiation therapy, or placebo manipulation (11–13).

Individuals experiencing cervical discomfort often exhibit restricted movement capabilities in the cervical spine and diminished muscular stamina in the neck region, significantly impairing their routine activities and potentially disrupting academic or occupational performance (14). Research indicates that rotational impairments in the neck area correlate more strongly with functional limitations compared to movement restrictions in other planes (15). Evaluation of the patient’s recovery is very important, as it is known that cervical mobility restriction is often associated with neck pain (16). In fact, available kinematic data suggest that the upper thoracic vertebra is also critical for overall cervical motion (17).

Numerous research investigations have demonstrated the therapeutic benefits of cervical spine manipulation (CSM) for alleviating discomfort in the neck region and enhancing cervical range of motion (CROM) among patients suffering from neck-related issues (18–20). Nevertheless, given the complex network of nerves and blood vessels in the cervical area, this therapeutic intervention presents potential hazards. These may include aggravated neck discomfort, cephalalgia, temporary neurological symptom exacerbation, and in rare instances, vertebral artery damage following CSM procedures (21). In recent years, numerous studies have attempted to treat neck pain patients through TSM, often demonstrating positive “immediate effects” after a single treatment session. This “immediate effect” of TSM on neck pain treatment typically refers to the changes in cervical pain intensity and range of motion immediately following a single treatment session. Its mechanisms of action are generally explained from the following aspects: On one hand, the cervicothoracic transition zone represents a biomechanical interface between the mobile cervical spine with its characteristic lordotic curve and the relatively rigid thoracic spine exhibiting kyphotic curvature, making this region particularly prone to stiffness (22). Reduced mobility at the cervicothoracic junction is thought to cause neck pain, headache, and upper limb pain (23). Research has identified abnormal movement patterns in the cervicothoracic region as being correlated with neck pain, highlighting the importance of restoring normal mobility in this area for affected individuals (24). Therapeutic approaches aimed at enhancing. The cervicothoracic junction plays a crucial role in minimizing motion requirements for the mid and lower cervical regions, consequently alleviating stress on the cervical vertebrae (25). This technique applied to the thoracic vertebrae and their junction with the cervical spine can immediately improve cervical range of motion, reduce pain, and restore optimal biomechanical status (26). On the other hand, the immediate pain relief effect produced by TSM is attributed to the inhibitory mechanism of descending stimulation. Walser et al.’s study demonstrated that manual stimulation can activate low-threshold mechanoreceptors (Aβ fibers), thereby inhibiting nociceptive transmission (C fibers) through the “gate control” mechanism (27).

Considering the anatomical characteristics of the cervicothoracic region, certain scholars have explored the use of thoracic spinal manipulation (TSM) as a safer intervention to alleviate cervical discomfort (10, 13). However, these studies primarily investigated and reported the improvement of cervical pain through TSM techniques, without covering changes in cervical range of motion across all directional planes or analyzing efficacy differences of different manipulation techniques in neck pain treatment through subgroup analyses. In other words, although multiple investigations have demonstrated short-term benefits of TSM for individuals experiencing neck pain (10, 18, 28), the medical community has not yet reached a definitive consensus or established standardized clinical guidelines. Moreover, some studies have reported different findings (29, 30), indicating that TSM failed to produce immediate pain relief effects in neck pain patients. To address this clinical uncertainty, we performed a systematic review of available randomized controlled trials to evaluate the short-term effectiveness of TSM in managing cervical pain and to offer scientific support for its therapeutic use.

## Methods

### Study design and registration

The study protocol for this meta-analysis was prospectively registered on the PROSPERO database (registration number CRD420261281887). The methodology for conducting this systematic review strictly adhered to the PRISMA framework (Preferred Reporting Items for Systematic Reviews and Meta-Analyses), as outlined in Liberati et al. (31).

### Data sources and searches

The research team developed comprehensive search strategies to identify relevant literature across multiple academic databases. In conducting a comprehensive literature review for this study, two investigators (YYP and JWC) systematically examined five major academic databases (PubMed, Embase, Web of Science, PEDro, and Cochrane Library). The search encompassed all available records from each database’s inception through January 30, 2023. A combination of MeSH and terms was used for the search, the subject terms were “thoracic spine manipulation,” “neck pain,” “immediate effect” and “randomized controlled trial,” and the subtopics were “cervical Pain,” “neck ache “, “cervical spine disease,” “neck spondylosis,” “spine manipulation,” “thoracic spine relaxation,” “spine relaxation” and “the Immediate effect.” A specific search strategy was used for each database, see Appendix. Furthermore, the team thoroughly examined existing systematic reviews and meta-analyses to identify potentially relevant randomized controlled trials that might have been incorporated in these secondary sources.

### Inclusion and exclusion criteria

To ensure comprehensive literature evaluation, predetermined selection and rejection standards were implemented. The inclusion requirements specified: (1) adult participants diagnosed with cervical pain; (2) intervention groups undergoing TSM therapy; (3) comparison groups receiving conventional cervical treatment or placebo TSM; (4) assessment of therapeutic outcomes using validated measurement tools including Visual Analogue Scale (VAS), Numeric Pain Rating Scale (NPRS), Cervical Range of Motion (CROM), and Neck Disability Index (NDI);

Exclusion criteria: (1) other causes of neck pain, such as healed neck trauma or generalized pain where the main pain point is not in the neck; (2) experimental and control groups received inconsistent treatments other than TSM; (3) unavailable complete manuscripts; (4) missing or duplicated data; (5) literature published in languages other than English.

### Study selection

The primary investigator (YYC) utilized Endnote 20 (Clarivate, Philadelphia, PA, United States) to compile all identified research papers within its document organization platform, where automated tools were employed to eliminate redundant entries. Subsequently, two independent evaluators (YL and ZCC) conducted parallel assessments by examining article titles and summaries, applying predetermined selection parameters during this initial phase. For further screening, two reviewers will download the full text and read through it, removing articles that do not meet the inclusion criteria and discussing them to confirm their eligibility. In instances where screening decisions proved contentious, resolution was achieved through consultation with the lead researcher (JL).

### Data extraction

Two evaluators (YYP and ZCC) autonomously collected the specified information from the selected randomized controlled trials, including the primary researcher’s name, publication year, participant count, patient demographics (age and sex), cervical pain classification, illness duration, therapeutic approaches employed, and measured endpoints. In instances where ambiguities or complexities arose during the data collection process regarding incomplete study information, the research team made efforts to reach out to the corresponding authors for clarification. Studies were classified as having incomplete data if no reply was obtained after three separate email attempts. Any discrepancies that emerged during the information gathering phase were adjudicated by a third researcher (JL).

### Risk of bias

Two independent investigators (ZCC and YL) performed the evaluation of literature quality, subsequently engaging in consensus discussions to ensure uniformity in findings. The Cochrane Risk of Bias instrument (Review Manager 5.4) was employed to examine potential biases across seven key dimensions: randomization procedures, concealment of allocation, blinding of study participants and staff, blinding of outcome evaluators, handling of missing data, selective reporting practices, and other potential sources of bias. Each potential bias was categorized as presenting either minimal risk, significant risk, or indeterminate risk.

The GRADE methodology (32, 33) was utilized to evaluate the strength of evidence, considering four principal factors: methodological constraints, indirect evidence, variability in findings, and measurement inaccuracies. Based on these criteria, the evidentiary value was classified into four tiers: strong, intermediate, limited, or minimal reliability (33).

### Statistical analysis

RCTs that used the same outcome indicators were pooled together for data analysis. According to the Cochrane Handbook (34), it is recommended that the value of the change between the baseline mean before the experimental intervention and the mean after the intervention be extracted as the difference in effect arising from the experimental procedure. The mean and standard deviation of differences, as well as sample size, were input into the statistical software. If difference values were not directly available and after contacting the authors without a response, they were calculated using Equation 1, where SD(b), SD(f), and SD(d) represent the standard deviation of baseline, final, and difference values, respectively, with a correlation coefficient R value estimated at 0.8. If the standard error (SE) was provided in the article, after contacting the author to obtain the standard deviation without getting a response, the standard deviation was calculated according to Equation 2, where *n* represents the sample size. If the median was provided in the article, the median was considered as the mean value. Extracted study data were entered into RevMan (v5.4, Copenhagen, Denmark) for statistical and analytical purposes. In addition, RCTs using uniform outcome indicators but with differences in intervention parameters in the experimental group were subjected to subgroup analysis to further analyze and compare intervention parameters with more therapeutic advantages. The Revman 5.4 software was employed to assess study variability, with the degree of heterogeneity quantified through the *I*^2^ statistic. Significant variability was defined as *I*^2^ values exceeding 75%, moderate variability as values between 50–75%, minimal variability as values ranging from 25–50%, and negligible variability for values below 25%.

The presence of heterogeneity was assessed using *I*^2^ statistics, with values below 0% indicating homogeneity (35). For all statistical evaluations, significance was determined at *p* < 0.05, accompanied by 95% confidence intervals (CIs).


(1) $$\mathrm{SD}(\text{d})=\sqrt{\mathrm{SD}(b)\;2+\mathrm{SD}(f)\;2-(2\times R\times \mathrm{SD}(b)\times \mathrm{SD}(f))\;}$$

(2) $$\mathrm{SD}(\text{d})=\mathrm{SE}\sqrt{n\;}$$

## Results

### Study search results

**Figure 1:**
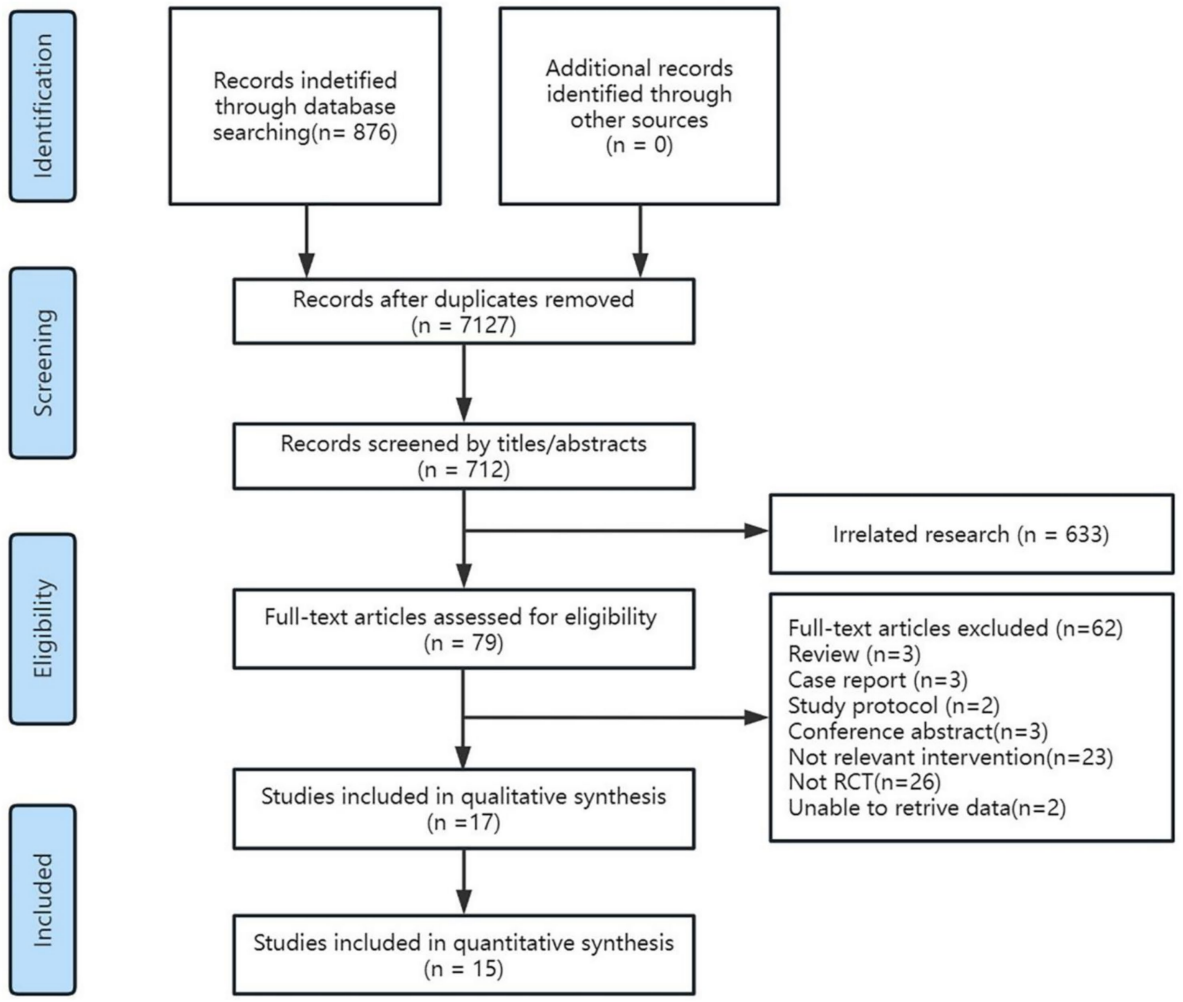
PRISMA flow diagram of literature search and study selection process.

Our initial literature search yielded 876 studies in total. The Endnote 20 software was used to import these studies, and 164 duplicates were screened. After reading all the literature’s titles and abstracts, 633 non-compliant studies were eliminated. After downloading and thoroughly reading the remaining 79 studies, 62 studies (26 non-randomized controlled trial studies, 34 studies devoid of pertinent interventions, and 2 studies for which data extraction was not possible) were still excluded. Finally, a total of 17 RCTs (12, 18, 23, 29, 30, 36–45) qualified for inclusion in the final analysis (Figure 1).

### Characteristics of included RCTs

Of the 17 RCTs we included, 12 RCTs (12, 18, 20, 29, 30, 36, 37, 39, 42, 44–46) examined the therapeutic outcomes of TSM versus placebo TSM, while three trials (23, 40, 46) evaluated the comparative effectiveness between TSM and CSM. Additionally, three studies (29, 42, 44) focused on comparing the efficacy of different TSM techniques. The detailed patient selection criteria for individuals suffering from cervical pain are presented in Table 1. The pooled data encompassed 1,100 participants with neck discomfort from eight different nations, with individual study populations varying between 27 and 107 subjects. While most participants were diagnosed with mechanical neck pain, there was significant variation in symptom duration across studies, including cases with acute symptoms (less than six weeks) and chronic conditions (exceeding six months), as documented in Table 2.

**Table 1:** Inclusion and exclusion criteria by study.

Study	Inclusion criteria	Exclusion criteria
Cleland et al. (36)	Participants were patients between 18 and 60 years of age with a primary complaint of referred by their primary care physician to an outpatient orthopaedic physical therapy clinic. Defined as nonspecific pain in the area of the cervicothoracic junction that is exacerbated by neck movements.	Patients with “red flags” for a serious spinal condition (e.g., infection, tumors, osteoporosis, spinal fracture, etc.) Pregnant Exhibited positive neurologic signs or symptoms suggestive of nerve root History of cervical or thoracic surgery Exhibited hypermobility of the thoracic spine Prior experience with spinal manipulative techniques
Cleland et al. (11, 12)	Ages of 18 and 60 years Have a primary complaint of neck pain with or without unilateral upper-extremity symptoms A baseline NDI score of 10% or greater	Identification of any medical signs suggestive of a nonmusculoskeletal etiology of symptoms History of a whiplash injury within 6 weeks of the examination Diagnosis of cervical spinal stenosis, evidence of any CNS involvement Signs consistent with nerve root compression (at least 2 of the following had to be diminished for nerve root involvement myotomal strength, sensation, or reflexes) Previous cervicothoracic surgery, or pending legal action
Krauss et al. (37)	Patients between 19 and 50 years old Presenting with complaints of non-traumatic posterior mid-cervical pain of an insidious onset in the region of the fourth to seventh cervical vertebral levels and aggravated with active cervical rotation were invited to participate	Patients with symptoms originating from the thoracic spine, systemic disease or autoimmune disease affecting the musculoskeletal system Positive radicular signs Myelopathy Previous surgery to the cervical spine
Lau et al. (38)	Age ranged between 18 and 55 with a diagnosis of mechanical neck pain for more than 3 months	Contraindication to manipulation History of whiplash or cervical surgery, diagnosis of fibromyalgia syndrome Undergone spinal manipulative therapy in the previous 2 months Loss of standing balance
Sillevis and Cleland (29)	Ages of 18 and 65 years and able to speak and read the English language fluently All subjects were instructed not to take any medication that could alter the functioning of the autonomic nervous system for at least 24 h before participating in the study Subjects were instructed not to consume caffeinated drinks, smoke, or eat anything for at least 12 h before the study	Diagnosed with autonomic diseases such as the Horner syndrome History of current neurological, ocular, and/or retinal disease Used 2 or more alcoholic beverages daily Trained for endurance sports
Dunning et al. (39)	Patients had to present with a primary complaint of neck pain (defined as pain in the region between the superior nuchal line and first thoracic spinous process) of any duration Between 18 and 70 years of age Have a NDI score of 20% or greater (10 points or greater on a 0-to-50 scale)	Exhibited any red flags (tumor, fracture, metabolic diseases, rheumatoid arthritis, osteoporosis, resting blood pressure greater than 140/90 mmHg, prolonged history of steroid use, etc.), presented with 2 or more Positive neurologic signs consistent with nerve root compression (muscle weakness involving a major muscle group of the upper extremity, diminished upper extremity deep tendon reflex, or diminished or absent sensation to pinprick in any upper extremity dermatome) Presented with a diagnosis of cervical spinal stenosis, exhibited bilateral upper extremity symptoms, Had evidence of CNS involvement (hyperreflexia, sensory disturbances in the hand, intrinsic muscle wasting of the hands, unsteadiness during walking, nystagmus, loss of visual acuity, impaired sensation of the face, altered taste, the presence of pathological reflexes) History of whiplash injury within the previous 6 weeks Had prior surgery to the neck or thoracic spine Had received treatment for neck pain from any practitioner within the previous month, Had pending legal action regarding their neck pain
Martínez-segura et al. (40)	Any signs of vertebrobasilar insufficiency (e.g., nystagmus, gait disturbances, Horner syndrome). Upper cervical spine ligamentous in stability through the Sharp-Purser test, alar ligament stress test, and transverse ligament test	Any contraindication to manipulation (e.g., positive extensionrotation test) Whiplash injury Previous cervical surgery Cervical radiculopathy or myelopathy Diagnosis of fibromyalgia Spinal manipulative therapy in the previous 6 months Younger than 18 or older than 65 years of age
Masaracchio et al. (41)	Between 18 and 60 years of age and to have neck pain without symptoms distal to the shoulder Pain of less than 3 months in duration A baseline NDI score of at least 20%	Any serious pathology (e.g., neoplasm, fracture) History of whiplash injury within the past 6 months A diagnosis of cervical spinal stenosis, unilateral or bilateral upper extremity radicular symptoms, Evidence of CNS involvement Evidence of nerve root compression Prior surgery to the cervical or thoracic spine Inability to speak English Any pending legal action Workers’ compensation or no-fault claims Being currently pregnant, or being unable to comply with treatment and follow-up guidelines
Suvarnnato et al. (42)	Aged 18 to 60 years; VAS pain rating of greater than or equal to 40 points; Symptoms of more than 3 months in duration Subject baseline ratings of neck pain were captured using a 100-point VAS with values ranging from 0 to 100.	Diagnosis of cervical radiculopathy or myelopathy (determined by a physiatrist) Previous history of cervical and thoracic spine fracture and/or dislocation; Previous history of surgery of the cervical and/or thoracic spine Previous history of spinal osteoporosis, spinal infection or fibromyalgia syndrome Previous history of underlying hypertension, heart disease or meningitis Pregnancy Any contraindication to manipulation History of SMT before this study
Casanova-Méndez et al. (43)	Aged between 18 and 60 years A minimum of a 3-month history of NSNP No minimum intensity of pain was specified NP not to be due to any known cause, such as fracture or infection Cervical pain was present with increased pain on one of the following criteria; with maintained posture, with movement and/or with palpation of the spinal muscles Perceived discomfort with joint pressure	Current use of any medication which might interfere with SMT The presence of any inflammatory disease Any neurological conditions Any bone pathology or history of tumors; Whiplash injury Having received SM in the previous 2 months Two or more positive signs of compressed nerves (changes in sensation, myotomal weakness in the arms, or alteration in deep tendon reflexes) Previous spinal surgery Any contraindication to SM Subjects who did not achieve cavitation after two thrust attempts
Salom-moreno et al. (44)	Neck pain symptoms of mechanical nature Age from 18 to 60 years Bilateral symptoms Symptoms for at least 6 months of duration.	Whiplash injury Previous spine surgery Diagnosis of cervical radiculopathy or myelopathy, Diagnosis of fibromyalgia, Having undergone any Physical therapy intervention in the previous year Pregnancy
Pires et al. (30)	Female university students between 18 and 39 years of age Pain or fatigue in the cervical region during activities of daily living or at rest for at least 6 months Diagnosis of neck pain based on the NDI	NDI score less than 5 points Body mass index greater than 25 kg/m^2^ Use of medication that can affect the musculoskeletal system (analgesic, anti-inflammatory, and muscle relaxer) Any sign of malignant tumor, inflammatory disease, or infectious condition that contraindicates the use of manual therapy History of whiplash History of surgery of the cervical spine Experience with spinal manipulation in the previous 2 months
Bautista-aguirre et al. (26)	Negative response to the Spurling test A positive response to the upper limb neurodynamic test of the median nerve in at least one upper extremity (both upper limbs were tested)	MNP secondary to whiplash, torticollis, rheumatoid arthritis, advanced cervical osteoarthritis and/or myelopathy Pprevious history of ischemic episodes Severe trauma or surgery in the upper extremity and/or cervical spine Carpal tunnel syndrome, hormonal imbalance, diabetes, or cervicobrachial pain associated with herniation or disc protrusion at lower cervical spine (T1-weighted sagittal and axial MRI images, as assessed by an independent physician)
Young et al (18)	18–65 yrs., NDI score ≥ 10/50 points, Diagnosis of cervical radiculopathy as defined by using 3 of 4 positive tests (Spurling’s test, upper limb neurodynamic test-median nerve bias, cervical distraction test, and cervical rotation towards the symptomatic side <60°)	History of previous cervical or thoracic spine surgery Bilateral upper extremity symptoms, signs or symptoms of upper motor neuron disorder, medical “red flags” (eg, tumor, fracture, rheumatoid arthritis, osteoporosis, prolonged steroid use) Cervical steroidal injection or medication within the past 2 weeks
Joshi et al. (23)	Age of 18 to 60 years Neck pain individuals with moderate to severe pain intensity, i.e., ≥4/10 on a NPRS with cervicothoracic junction dysfunction Subjects with pain provocation and reduced mobility at the CT junction segment	History of recent significant trauma, previous spine surgery, presence of any red flags, or pregnancy. Neck pain was associated with cervical radiculopathy, whiplash injuries, severe headaches, cervical spine fracture, or vertebrobasilar insufficiency
Erdem et al. (20)	Male patients between 18–25 years Having chronic or recurrent neck or shoulder pain (between 3–5 out of 10) at least 3 months duration with or without arm pain	Cervical disc herniation, vertebrobasilar insufficiency, systemic or neurological diseases, orthopedic problems associated with cervical or/and shoulder Joints History of trauma or surgery of the vertebral colon or shoulder
Romero Del Rey et al. (46)	Nystagmus Gait disturbances or Horner’s syndrome Underwent screening for upper cervical spine ligamentous instability through Sharp-Purser test, alar ligament stress test and transverse ligament tests	Contraindication to cervical thrust joint manipulation (e.g., fracture, osteoporosis, positive extension-rotation test or any symptom of vertebrobasilar insufficiency) History of whiplash History of cervical spine Surgery Diagnosis of cervical radiculopathy or Myelopathy Diagnosis of fibromyalgia syndrome Having previously undergone SMT Being less than 18 or more than 55 years of age

MNP, mechanical neck pain; NDI, neck disability index; CNS, central nervous system; SMT, spinal manipulative therapy; NPRS, numeric pain rating scale.

**Table 2:** Participant characteristics by study.

Study	Participants	Interventions	Outcome measure	Main results	Adverse events
Cleland et al. (36)	*n* = 27; 0 male, 27 female; mean ± SD age, symptom duration, 36 ± 8.5y; 12.2 ± 3.5wk Diagnosis: patient with MNP	Group 1 (*n* = 14): TSM Group 2 (*n* = 13): Placebo TSM	Pain (at night and in the day, with and without motion) (10-cm VAS) Disability (0–50 NDI)	Statistically significant differences between both groups (*p* < 0.05) Not reported	No
Cleland et al. (11, 12)	*n* = 60; 27 male, 33 female; mean ± SD age, 43.8 ± 11.5 y; symptom duration, 54.9 ± 46.0d Diagnosis: patient with NP	Group 1 (*n* = 30): TSM Group 2 (*n* = 30): Placebo TSM	Pain (at night and in the day, with and without motion) (10-cm NPRS) Disability (0–50 NDI)	Statistically significant differences between both groups (*p* < 0.05)*	Muscle spasm Headache
Krauss et al. (37)	*n* = 32; proportion not reported; mean ± SD age, proportion not reported; symptom duration, proportion not reported; Diagnosis: patient with MNP	Group 1 (*n* = 16): TSM Group 2 (*n* = 16): Placebo TSM	Pain (at night and in the day, with and without motion) (10-cm VAS) CROM (flexion, extension, left lateral flexion, right lateral flexion, right rotation, left rotation) (deg)	Statistically significant differences between both groups (*p* < 0.05)*	No
Lau et al. (38)	*n* = 120; 60 male, 60 female; mean ± SD age, 44.17 ± 9.27y; symptom duration, proportion not reported; Diagnosis: patient with MNP	Group 1 (*n* = 60): TSM + IRR Group 2 (*n* = 60): IRR	Pain (at night and in the day, with and without motion) (10-cm NPRS) ROM (flexion, extension, left lateral flexion, right lateral flexion, right rotation, left rotation) (deg)	Statistically significant differences between both groups (*p* < 0.05)*	No
Sillevis and Cleland (29)	*n* = 100; 23 male, 77 female; mean ± SD age, 42.70y; symptom duration, 23.3; Diagnosis: patient with MNP	Group 1 (*n* = 50): TSM (thrust) Group 2 (*n* = 50): TSM (non–thrust mobilization)	Pain (at night and in the day, with and without motion) (10-cm VAS)	Statistically significant differences between both groups (*p*>0.05)	-
Dunning et al. (39)	*n* = 107; 34 male, 73 female; mean ± SD age, 41.5 ± 11.9y; symptom duration, 336.9 ± 527.7d Diagnosis: patient with MNP	Group 1 (*n* = 56): TSM Group 2 (*n* = 51): Placebo TSM	Disability (0–50 NDI) Pain (at night and in the day, with and without motion) (10-cm NPRS)	Statistically significant differences between both groups (*p* < 0.05)*	No
Martínez-segura et al. (40)	*n* = 90; 44 male, 46 female; mean ± SD age, 38 ± 7y; symptom duration, 3.8 ± 1.5y Diagnosis: patient with CMNP	Group 1 (*n* = 33): TSM Group 2 (*n* = 57): CSM	CROM (flexion, extension, left lateral flexion, right lateral flexion, right rotation, left rotation) (deg) PPT	Statistically significant differences between both groups (*p* < 0.05)*	Neck fatigue Neck pain
Masaracchio et al. (41)	*n* = 66; 16 male, 50 female; mean ± SD age, 30.5 ± 9.5y; symptom duration, 37.3 ± 25.3d Diagnosis: patient with MNP	Group 1 (*n* = 34): TSM + exercise Group 2 (*n* = 32): exercise	Disability (0–50 NDI) Pain (at night and in the day, with and without motion) (10-cm NPRS)	Statistically significant differences between both groups (*p* < 0.05)*	No
Suvarnnato et al. (42)	*n* = 39; 11 male, 28 female; mean ± SD age, 37 ± 12.5y; symptom duration, more than 3 m Diagnosis: patient with CMNP	Group 1 (*n* = 13) TSM (thrust) Group 2 (*n* = 13) TSM (non–thrust mobilization) Group 3 (*n* = 13) Placebo TSM	Pain (at night and in the day, with and without motion) (10-cm VAS) CROM (flexion, extension, left lateral flexion, right lateral flexion, right rotation, left rotation) (deg)	Statistically significant differences between both groups (*p* < 0.05)*	No
Casanova-Méndez et al. (43)	*n* = 60; 17 male, 43 female; mean ± SD age, 37.5 ± 9.4y; symptom duration, more than 3 m Diagnosis: patient with CMNP	Group 1 (*n* = 30) TSM-DT Group 2 (*n* = 30) TSM-TRT	Pain (at night and in the day, with and without motion) (10-cm VAS) CROM (flexion, extension, left lateral flexion, right lateral flexion, right rotation, left rotation) (deg) PPT	Statistically significant differences between both groups (*p* < 0.05)*	No
Salom-moreno et al. (44)	*n* = 52; 30 male, 22 female; mean ± SD age, 32 ± 7y; symptom duration, 2.2 ± 1.1y Diagnosis: patient with CMNP	Group 1 (*n* = 27): TSM (thrust) Group 2 (*n* = 25): TSM (non–thrust mobilization)	Pain (at night and in the day, with and without motion) (10-cm VAS) PPT	Statistically significant differences between both groups (*p* < 0.05)*	Cervicothoracic discomfort
Pires et al. (30)	*n* = 32; 0 male, 32 female; mean ± SD age, 23.5 ± 4y; symptom duration, more than 6 m Diagnosis: patient with CNP	Group 1 (*n* = 56): TSM Group 2 (*n* = 51): Placebo TSM	Pain (at night and in the day, with and without motion) (10-cm VAS) Disability (0–50 NDI)	Statistically significant differences between both groups (*p*>0.05)	No
Bautista-aguirre et al. (26)	*n* = 32; 22 male, 66 female; mean ± SD age, 31.13 ± 6.15y; symptom duration, more than 12 wks Diagnosis: patient with CMNP	Group 1 (*n* = 30) CSM Group 2 (*n* = 28) TSM Group 3 (*n* = 30) Placebo TSM	PPT	Statistically significant differences between both groups (*p* < 0.05)*	No
Young et al. (18)	*n* = 43; 14 male, 29 female; mean ± SD age, 18–65y; symptom duration, not reported Diagnosis: patient with cervical radiculopathy	Group 1 (*n* = 22): TSM Group 2 (*n* = 21): Placebo TSM	Pain (at night and in the day, with and without motion) (10-cm NPRS)	Statistically significant differences between both groups (*p* < 0.05)*	No
Joshi et al. (23)	*n* = 42; 23 male, 19 female; mean ± SD age, 35.1 ± 10.1y; symptom duration, not reported Diagnosis: patient with MNP	Group 1 (*n* = 21): TSM Group 2 (*n* = 21): CJM	Pain (at night and in the day, with and without motion) (10-cm NPRS)	Statistically significant differences between both groups (*p* < 0.05)*	No
Erdem et al. (20)	*n* = 80; 50 male, 30 female; mean ± SD age, 20 ± 4.9y; symptom duration, more than 6 m Diagnosis: patient with MNP	Group 1 (*n* = 50): TSM Group 2 (*n* = 30): Placebo TSM	CROM (flexion, extension, left lateral flexion, right lateral flexion, right rotation, left rotation) (deg)	Statistically significant differences between both groups (*p* < 0.05)*	No
Romero Del Rey et al. (46)	*n* = 82; 41 male, 41 female; mean ± SD age, 45 ± 8y; symptom duration, 83 ± 7 m Diagnosis: patient with CNP	Group 1 (*n* = 41): TSM + CJM Group 2 (*n* = 41): CJM	Pain (at night and in the day, with and without motion) (10-cm VAS) Disability (0–50 NDI) CROM (flexion, extension, left lateral flexion, right lateral flexion, right rotation, left rotation) (deg)	Statistically significant differences between both groups (*p* < 0.05)*	Increased neck pain Neck fatigue

*Values are mean ± SD where those data were provided by the authors. NDI, Neck Disability Index; NPRS, Numeric Pain Rating Scale; IRR, infrared radiation therapy; TSM, Thoracic spine manipulation; CSM, Cervical spine manipulation; DT, Dog technique; TRT, Toggle-recoil technique; CJM, Cervicothoracicjunction mobilization; CROM, Cervical range of motion; PPT, Pressure pain threshold.

**Figure 2:**
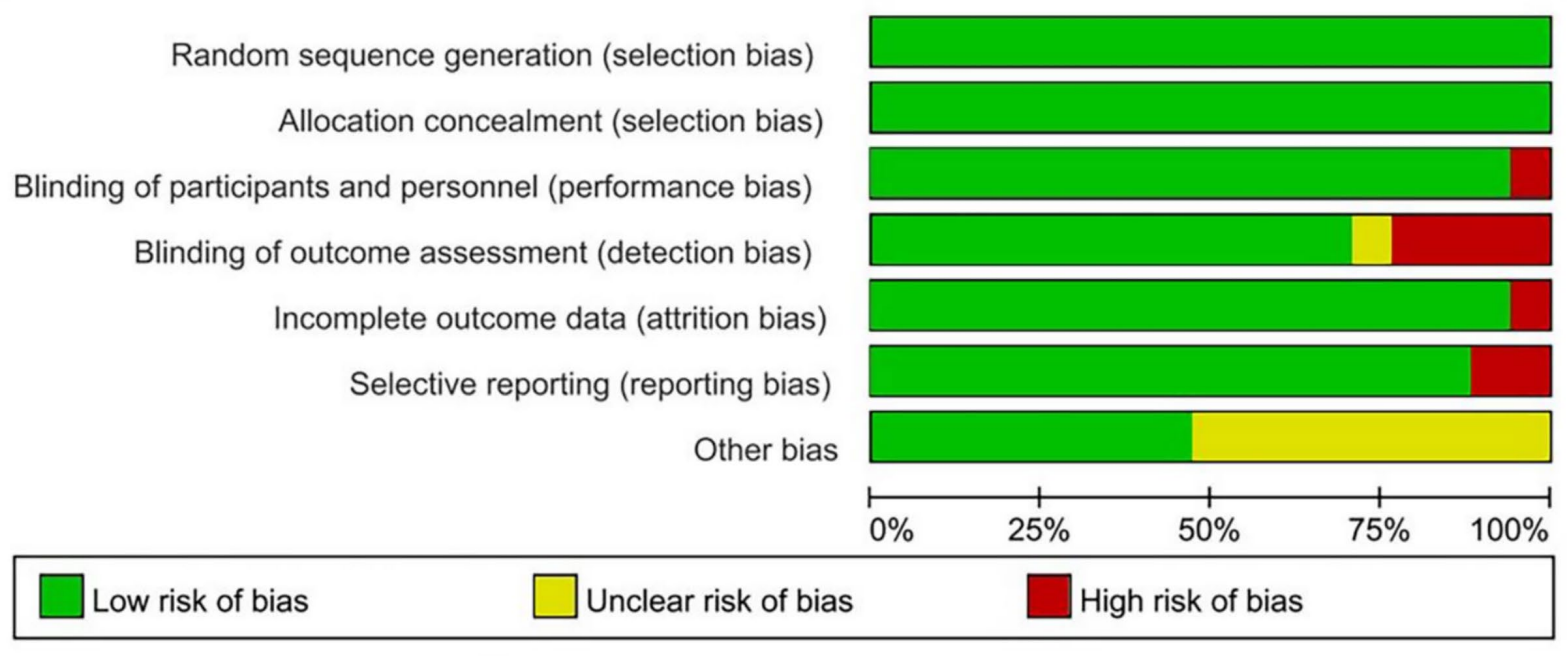
Risk of bias summary: review of authors’ judgments about each risk of bias item for all included studies.

**Figure 3:**
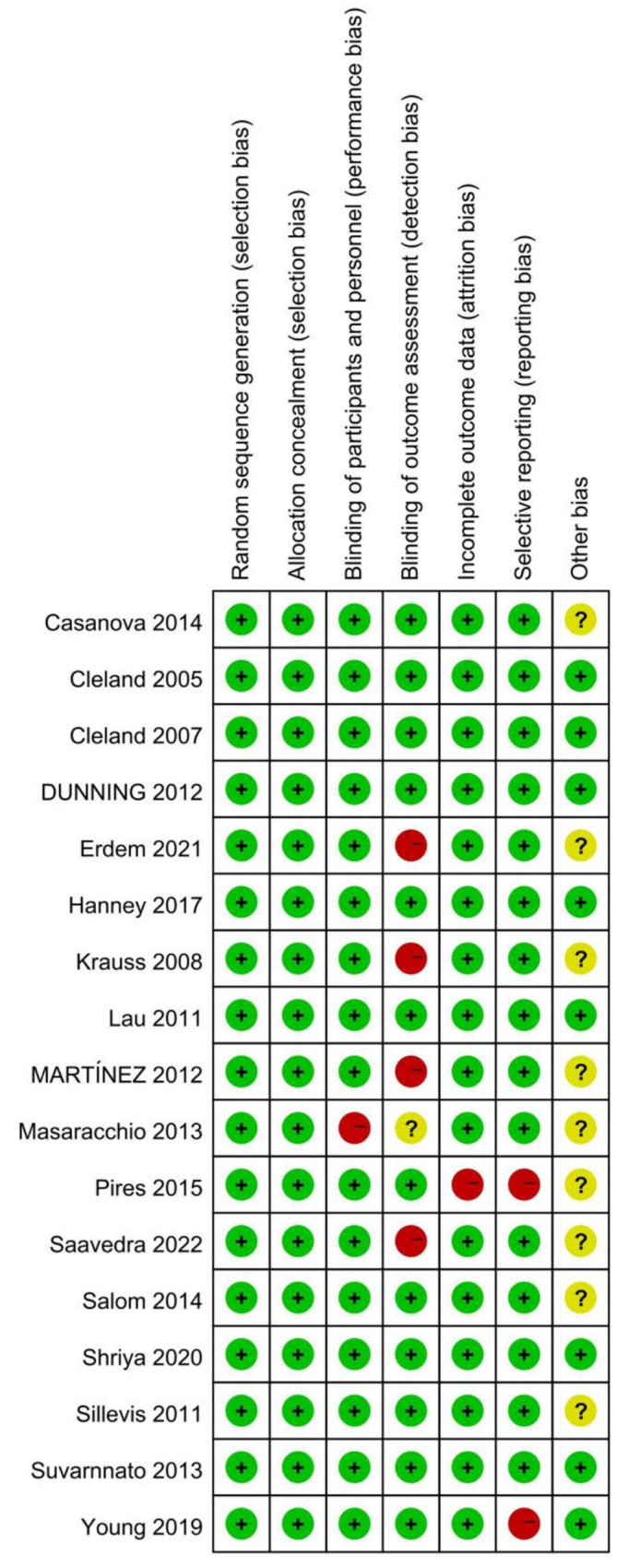
Risk of bias graph: percentage of studies with low, high, or unclear risk of bias across each domain.

An evaluation of methodological quality was conducted using the Cochrane Risk of Bias Tool (Revman 5.4), revealing several limitations in the included research. Four investigations (20, 37, 40, 46) exhibited elevated detection bias due to inadequate blinding procedures during effectiveness evaluation. Two trials (18, 30) demonstrated significant reporting bias as they failed to document all primary outcome measures comprehensively. Performance bias was identified in one study (41) owing to insufficient blinding of both participants and practitioners. Additionally, one trial (30) presented notable attrition bias because certain outcome data were incomplete. The remaining studies demonstrated minimal bias in methodological assessment, with the overall analysis indicating that the randomized controlled trials included in our review maintained satisfactory quality standards (Figures 2, 3).

Table 3 presents an overview of the evaluation findings concerning key performance measures, specifically the Visual Analog Scale (VAS), Numeric Pain Rating Scale (NPRS), Neck Disability Index (NDI), and Cervical Range of Motion (CROM). The data reveals superior quality evidence supporting the therapeutic advantages of TSM over placebo or CSM interventions in enhancing cervical mobility and reducing functional impairment. Regarding pain reduction, the supporting evidence was classified as intermediate in quality. Examination of other parameters showed no significant adverse effects or methodological limitations, with all remaining indicators maintaining high evidentiary standards. Consequently, the GRADE system assigned a “strong” recommendation rating to all four primary assessment criteria.

**Table 3:** Level of quality evidence for the effectiveness of TSM on neck pain.

Certainty assessment							No. of participants		Effect			
No. of studies	Study design	Risk of Bias	Inconsistency	Indirectness	Imprecision	Publication bias	TSM	Control	SMD [95% CI]	Size	Certainty	Direction
Pain intensity												
Outcome: VAS												
8	RCT	Not serious	Not serious	Not serious	Not serious	Not serious	176/347 (51%)	171/347 (49%)	−0.86 [−1.08, −0.63]	Moderate	⊕ ⊕ ⊕⊖ Moderate	In favor of TSM
Outcome: NPRS												
6	RCT	Not serious	Not serious	Not serious	Not serious	Not serious	223/438 (51%)	215/438 (49%)	−0.80 [−1.13, −0.46]	Moderate	⊕ ⊕ ⊕⊖ Moderate	In favor of TSM
Cervical range of motion												
Outcome: flexion												
5	RCT	Not serious	Not serious	Not serious	Not serious	Not serious	198/376 (53%)	178/376 (47%)	4.27 [2.14, 6.40]	Large	⊕ ⊕ ⊕⊕ High	In favor of TSM
Outcome: extension												
5	RCT	Not serious	Not serious	Not serious	Not serious	Not serious	198/376 (53%)	178/376 (47%)	2.33 [0.17, 4.49]	Large	⊕ ⊕ ⊕⊕ High	In favor of TSM
Outcome: right lateral flexion												
	RCT	Not serious	Not serious	Not serious	Not serious	Not serious	198/376 (53%)	178/376 (47%)	2.94 [1.09, 4.78]	Large	⊕ ⊕ ⊕⊕ High	In favor of TSM
Outcome: left lateral flexion												
	RCT	Not serious	Not serious	Not serious	Not serious	Not serious	198/376 (53%)	178/376 (47%)	2.29 [0.47, 4.10]	Large	⊕ ⊕ ⊕⊕ High	In favor of TSM
Outcome: right rotation												
	RCT	Not serious	Not serious	Not serious	Not serious	Not serious	198/376 (53%)	178/376 (47%)	2.47 [0.18, 4.76]	Large	⊕ ⊕ ⊕⊕ High	In favor of TSM
Outcome: left rotation												
	RCT	Not serious	Not serious	Not serious	Not serious	Not serious	198/376 (53%)	178/376 (47%)	3.15 [0.82, 5.47]	Large	⊕ ⊕ ⊕⊕ High	In favor of TSM
Disability												
Outcome: NDI												
4	RCT	Not serious	Not serious	Not serious	Not serious	Not serious	161/315 (51%)	154/315 (49%)	−7.31 [−10.01, −4.61]	Small	⊕ ⊕ ⊕⊕ High	In favor of TSM

RCT, randomized controlled trials; NDI, Neck Disability Index; TSM, Thoracic spine manipulation; CROM, Cervical range of motion.

## Effectiveness

### Effects of TSM on pain intensity

**Figure 4:**
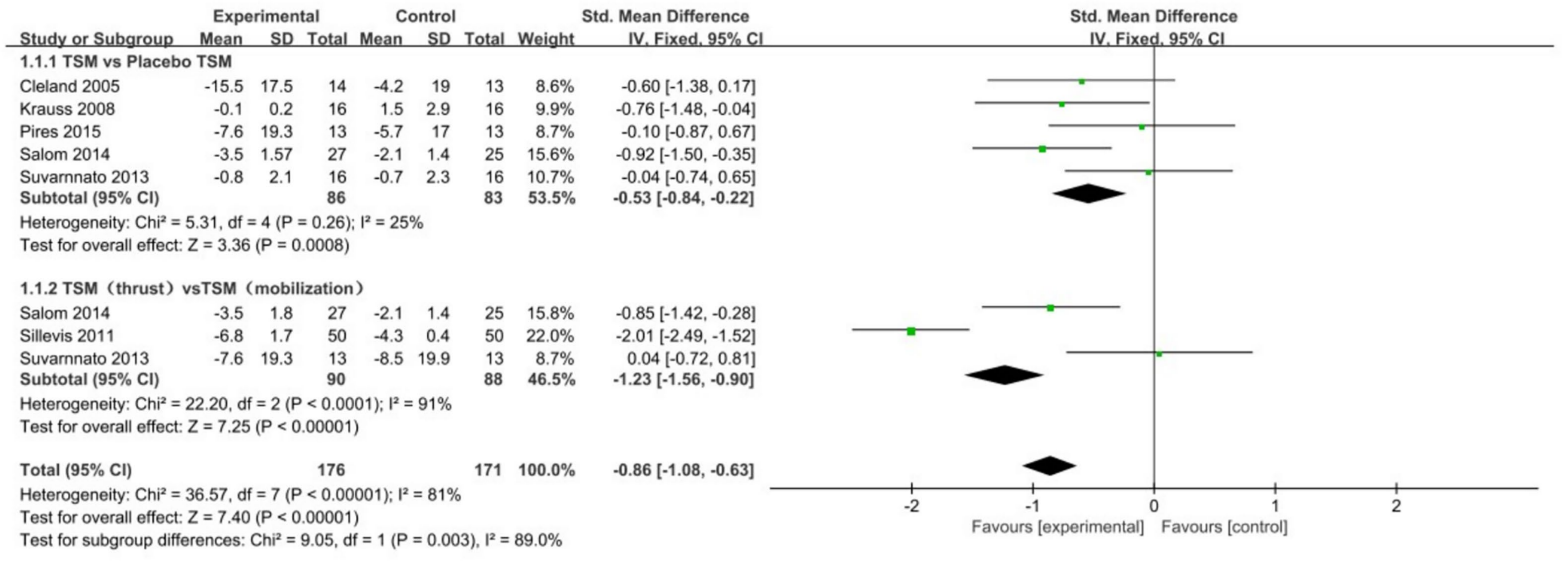
Forest plot comparing the effect of thoracic spine manipulation versus control on pain intensity (Visual Analogue Scale, VAS).

**Figure 5:**
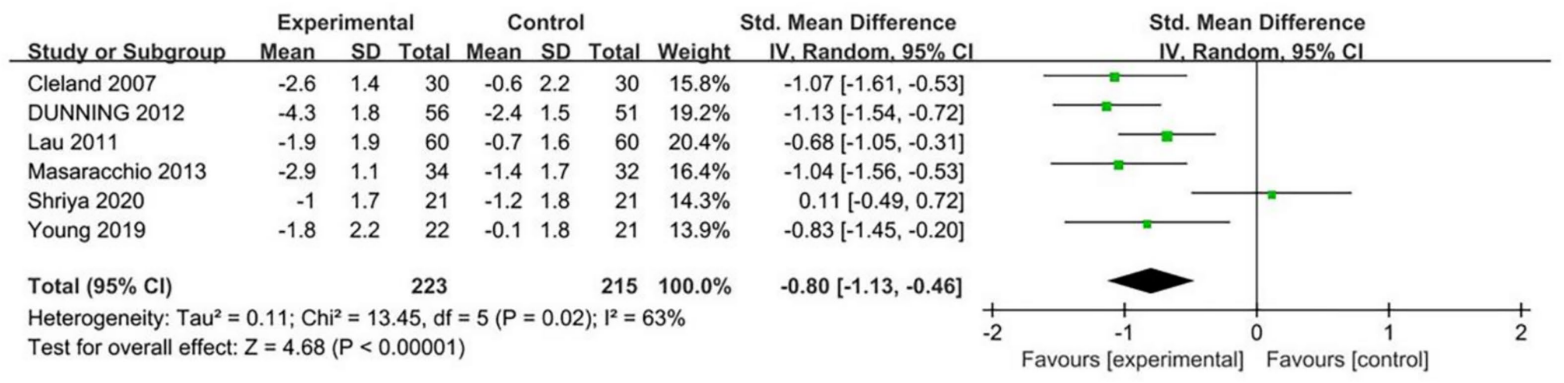
Forest plot comparing the effect of thoracic spine manipulation versus control on pain intensity (Numeric Pain Rating Scale, NPRS).

11 RCTs (18, 23, 30, 36–39, 41, 42, 44) assessed the intensity of neck pain in patients using the VAS or NPRS. When comparing the treatment group receiving TSM therapy with the control group, a statistically significant reduction in VAS scores was observed (Standardized Mean Difference = −0.53, 95% Confidence Interval: −0.84 to −0.22, *I*^2^ = 25%, *p* < 0.05, as illustrated in Figure 4). Notably, the thrust technique employed in TSM demonstrated greater efficacy in lowering VAS scores than non-thrust mobilization methods (SMD = -1.23, 95% CI: −1.56 to −0.90, *I*^2^ = 91%, *p* < 0.05, Figure 4). Furthermore, TSM intervention yielded significant improvements in NPRS measurements relative to control conditions (SMD = −0.80, 95% CI: −1.13 to −0.46, *I*^2^ = 63%, *p* < 0.05, Figure 5). These findings from both pain assessment scales collectively indicate that TSM therapy effectively alleviates pain symptoms in individuals suffering from neck discomfort.

### Effects of TSM on CROM

**Figure 6:**
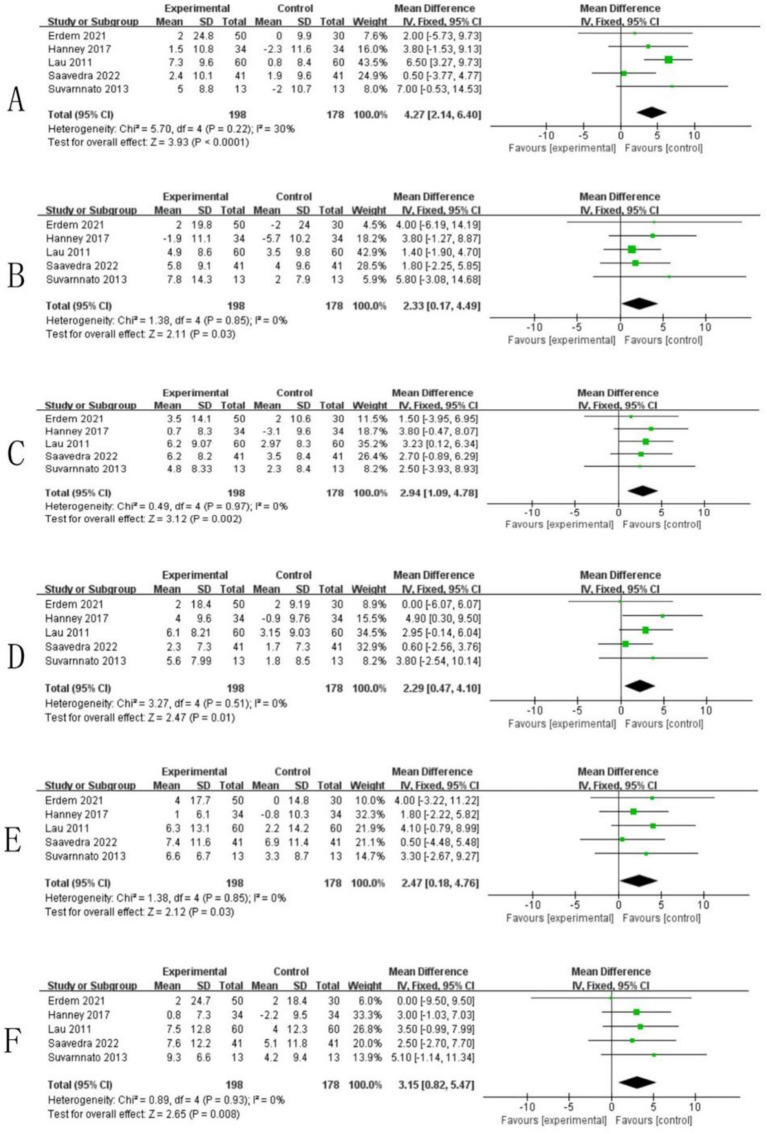
Forest plots comparing the effect of thoracic spine manipulation versus control on cervical range of motion (CROM). **(A)** Comparison of thoracic spine manipulation versus control on cervical forward flexion. **(B)** Comparison of thoracic spine manipulation versus control on cervical extension. **(C)** Comparison of thoracic spine manipulation versus control on cervical right lateral flexion. **(D)** Comparison of thoracic spine manipulation versus control on cervical left lateral flexion. **(E)** Comparison of thoracic spine manipulation versus control on cervical right rotation. **(F)** Comparison of thoracic spine manipulation versus control on cervical left rotation.

Five research studies (20, 38, 42, 45, 46) evaluated cervical range of motion (CROM) in individuals suffering from neck discomfort, conducting measurements both prior to and following the intervention. Meta-analysis through forest plot demonstrated that the TSM approach yielded statistically significant enhancements in CROM parameters when compared to control groups. The therapeutic effects manifested across all cervical movement planes, with quantitative outcomes as follows: flexion (standardized mean difference = 4.27, confidence interval = [2.14, 6.40], heterogeneity index = 30%, significance level<0.05, Figure 6A), extension (SMD = 2.33, CI = [0.17, 4.49], I2 = 0%, *p* < 0.05, Figure 6B), right side bending (SMD = 2.94, CI = [1.09, 4.78], I2 = 0%, *p* < 0.05, Figure 6C), left side bending (SMD = 2.29, CI = [0.47, 4.10], I2 = 0%, *p* < 0.05, Figure 6D), right twisting (SMD = 2.47, CI = [0.18, 4.76], I2 = 0%, *p* < 0.05, Figure 6E), and left rotation (SMD = 3.15, CI = [0.82, 5.47], I2 = 0%, *p* < 0.05, Figure 6F). Comprehensive analysis confirmed that TSM intervention produced clinically meaningful improvements in all measured dimensions of cervical mobility for neck pain sufferers.

### Effects of TSM on disability

**Figure 7:**
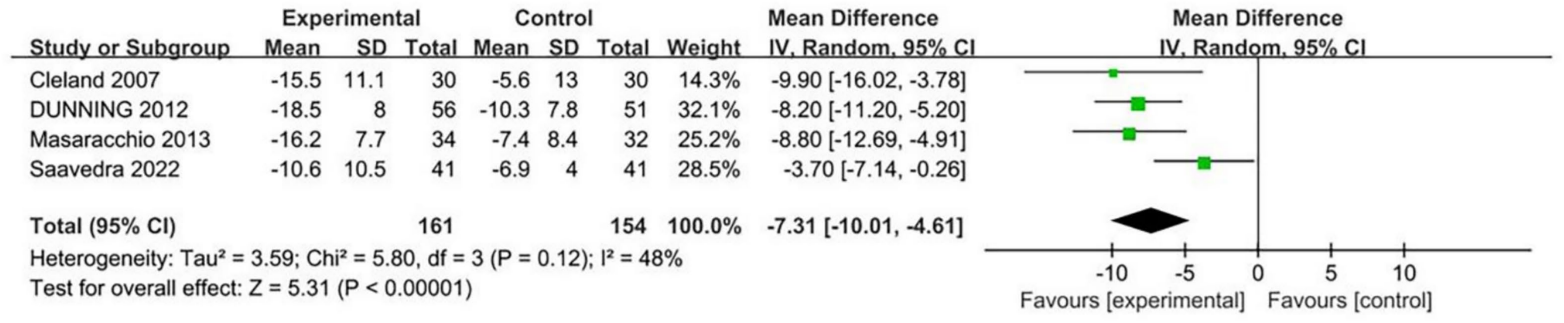
Forest plot comparing the effect of thoracic spine manipulation versus control on neck disability index (NDI) scores.

Four research investigations (12, 39, 41, 46) employed the Neck Disability Index (NDI) as an evaluation tool to measure cervical dysfunction severity among participants. When examining the forest plot data derived from NDI measurements, Traditional Spinal Manipulation (TSM) demonstrated superior efficacy in enhancing NDI outcomes for individuals suffering from cervical discomfort when contrasted with control groups (Standardized Mean Difference = −7.31, 95% Confidence Interval = [−10.01, −4.61], *I*^2^ = 48%, statistical significance *p* < 0.05, refer to Figure 7). The forest plot interpretation reveals that TSM intervention significantly contributed to reducing functional impairment levels in cervical pain patients.

Due to the limited number of included studies and their small sample sizes, we were unable to conduct subgroup analyses based on additional stratifications, such as population type, symptom duration, control interventions, dosage, technical standardization, or clinician experience. Therefore, we employed a random-effects model to mitigate the impact of high heterogeneity (*I*^2^ > 50%) on the results. Furthermore, we performed sensitivity analysis using the “leave-one-out” method for studies contributing to high heterogeneity. By iteratively excluding individual studies via Review Manager software, we observed that the final statistical results remained robust regardless of which study was removed (*p* values consistently remaining below 0.05).

### Adverse events

Among our included studies, 12 studies (12, 18, 23, 36–41, 43, 44, 46) that recorded treatment-related complications. Of these, 8 investigations (18, 23, 36–39, 41, 43) found no negative effects in cervical pain patients receiving TSM therapy. Conversely, the remaining 4 studies (12, 40, 44, 46) identified various treatment-related symptoms including muscular contractions, cephalalgia, cervical exhaustion, cervical discomfort, and upper back uneasiness among TSM recipients. Notably, the incidence of these complications was minimal, their persistence brief, and no participants discontinued the clinical trials due to these temporary adverse effects.

## Discussion

The findings of our study demonstrate that TSM therapy yields significant clinical benefits for individuals suffering from cervical pain. This therapeutic approach not only alleviates pain intensity as measured by both VAS and NPRS scales but also enhances cervical mobility. These improvements are reflected in decreased NDI values and expanded CROM measurements. Our investigation also revealed variations in treatment outcomes among different TSM techniques when applied to cervical pain patients. To better understand these findings, we conducted an in-depth examination of TSM’s working mechanisms for cervical pain management. Additionally, we performed comparative analyses between various TSM techniques and conventional CSM methods to evaluate their relative effectiveness.

The vertebral column consists of interconnected cervical, thoracic, lumbar, and sacral segments, with the cervical curvature maintaining a dynamic relationship with the thoracic curvature. Consequently, patients experiencing cervical discomfort often demonstrate altered thoracic biomechanics due to muscular tension in the neck region, and this reciprocal influence operates in both directions (47). During the late 20th century, Pinto and colleagues (48) emphasized the necessity of comprehensive assessment of both cervical and thoracic regions when managing cervical pain cases. Their research indicated that thoracic biomechanical disturbances inevitably affect cervical vertebral stress distribution, suggesting that therapeutic interventions focusing solely on the cervical area might prove ineffective without addressing thoracic dysfunction. To further investigate this biomechanical interdependence, Tsang et al. (49) conducted detailed three-dimensional motion analysis of spinal segments, a spinal sensor study revealed that individuals experiencing cervical discomfort consistently exhibited misalignment between their cervical and upper thoracic regions. Wang et al. (50) corroborated this observation through electromagnetic induction technology, analyzing movement patterns in these spinal segments. These findings clearly demonstrate the strong biomechanical interdependence between the cervical and thoracic regions. TSM manipulation techniques enhance thoracic mobility, thereby normalizing the biomechanical relationship within the cervical-thoracic movement complex (51). This intervention alleviates mechanical strain on cervical structures, consequently diminishing neck discomfort. Research further indicates that cervical pain frequently manifests with associated muscular symptoms, including tenderness and increased muscle tone in the neck area (52). Electronic pressure measurements conducted by researchers (53) demonstrated elevated pain sensitivity in hyperactive upper cervical muscles.

The development of neck and shoulder discomfort is frequently associated with excessive engagement of the upper trapezius muscle coupled with diminished function in both the lower trapezius and anterior serratus muscles. These muscle groups predominantly originate from the thoracic region of the spine. Therapeutic spinal manipulation (TSM) demonstrates rapid efficacy in alleviating cervical discomfort by promoting muscular relaxation in affected areas, consequently diminishing tension in cervical musculature and providing symptomatic relief.

Furthermore, TSM exerts mechanical stress on spinal structures including intervertebral discs, adjacent ligaments, and tendons. This mechanical stimulation enhances proprioceptive feedback, activating type I and II afferent nerve pathways. Such neural activation can trigger reflexive pain suppression or muscle relaxation responses (54), offering additional therapeutic benefits for individuals suffering from cervical pain.

Research conducted by Martínez-Segura et al. (40) and Joshi et al. (23) evaluated the comparative outcomes of thoracic spinal manipulation (TSM) and cervical spinal manipulation (CSM) for individuals suffering from neck discomfort. Their findings indicated that both therapeutic approaches demonstrated comparable effectiveness in alleviating symptoms associated with neck pain. Martínez’s investigation specifically revealed that patients experiencing acute neck pain showed no signs of developing tolerance to repeated TSM applications. This observation aligns with the theoretical framework proposed by Boal and Gillette’s research group (55), which posits that TSM maintains its ability to produce prolonged counter-stimulation effects while simultaneously inhibiting the body’s natural pain response mechanisms. Joshi’s parallel study further suggested that TSM’s benefits extend beyond mere structural adjustments, highlighting its capacity to induce positive neurophysiological changes that contribute to pain relief. While CSM remains particularly effective in directly addressing muscular tension and realigning cervical vertebrae in neck pain patients, thoracic manipulation techniques have also demonstrated significant therapeutic potential. Therapeutic outcomes comparable to CSM can be attained through TSM by rapidly diminishing the patient’s pain perception. This is accomplished by suppressing neural activity in the upper spinal cord areas responsible for central pain modulation, aligning with established research in chiropractic interventions (56). These findings demonstrate that TSM serves as a viable alternative to CSM for achieving rapid neck pain relief, particularly given CSM’s elevated procedural risks. The immediate analgesic effects observed suggest TSM’s clinical value in cervical pain management scenarios where safety considerations are paramount.

In the selected research literature, three investigations (29, 42, 44) evaluated the comparative effectiveness between thrust and non-thrust mobilization techniques within thoracic spine manipulation (TSM) for cervical pain management. Meta-analysis through forest plot demonstrated superior pain relief outcomes with thrust interventions compared to non-thrust approaches. The non-thrust method primarily involved articular compression accompanied by audible joint cavitation, yet failed to adequately address muscular tension or provide repeated mechanical correction and stress application to the thoracic region. Consequently, when considering both biomechanical and neurophysiological mechanisms, the therapeutic benefits of non-thrust mobilization prove substantially inferior to those achieved through thrust techniques in TSM protocols.

The vertebral regions encompassing the neck and upper back have been the focus of therapeutic interventions. Sillevis and Cleland (29) highlighted in his research that practitioners should avoid relying on joint cavitation sounds as a definitive marker of treatment efficacy when applying TSM manipulation to individuals experiencing cervical discomfort. Nevertheless, the evidence synthesized in our meta-analysis clearly demonstrates that both thrust-based and non-thrust mobilization approaches within TSM methodology yield rapid pain relief for those suffering from neck pain. While existing randomized controlled trials fail to provide conclusive data comparing the effectiveness of combined TSM techniques versus isolated thrust methods, the practical application of integrating both approaches in clinical settings appears promising. This finding underscores the potential value of further investigation into hybrid therapeutic strategies that incorporate both thrust and non-thrust mobilization components within TSM protocols.

TSM demonstrates significant clinical improvement effects in patients with neck pain, a finding that is also consistent with recent related studies. The study by Kocaman et al. (57) further confirmed that the Mulligan mobilization technique combined with cervical stabilization exercises is superior to cervical stabilization exercises alone in improving chronic neck pain, showing more significant therapeutic effects particularly in pain relief and functional recovery. Similarly, Ceylan et al. (58) compared the effectiveness of two different treatment approaches in a randomized controlled trial of patients with chronic non-specific neck pain, and the results showed that cervical mobilization exercises combined with conventional treatment could significantly improve patients’ pain, muscle strength, cervical range of motion, and anxiety status. The innovation of this study lies in the research team’s particular attention to changes in attention indicators, suggesting that interventions in the cervical region may have positive effects on cognitive function (especially in attention, memory, and concentration). Notably, existing studies have begun to focus on the potential benefits of manual therapy on cognitive function. The study by Ceylan et al. (58) suggests that cervical mobilization exercises may improve local blood perfusion and neural regulation, thereby positively affecting patients’ attention and anxiety status. Based on this, we speculate that TSM may produce broader beneficial effects on cognitive function in neck pain patients through similar neural mechanisms, including enhanced memory, improved attention, and better concentration. These cognitive improvements may stem from the following mechanisms: First, TSM-induced activation of the descending pain inhibitory system can reduce the occupation of cognitive resources by chronic pain, thereby releasing more cognitive capacity for memory encoding and information processing; second, improved proprioceptive input in the cervicothoracic region may enhance cortical arousal through the thalamus-cortical pathway, promoting attention concentration; finally, TSM-related autonomic regulation (such as reduced sympathetic tone) may optimize cerebral blood perfusion, providing a more favorable neurophysiological environment for higher cognitive functions. However, current research on the effects of TSM on cognitive function is still in its preliminary stage. Future studies should adopt standardized neuropsychological assessment tools (such as working memory tasks and sustained attention tests), combined with functional neuroimaging techniques (such as fNIRS or fMRI), to objectively quantify the improvement effects of TSM on cognitive function in neck pain patients. In addition, long-term follow-up studies will help determine the duration of these cognitive benefits and their association with clinical symptom improvement.

TSM demonstrates significant differences between immediate and long-term effects on neck pain. Immediate effects (within minutes to 72 h after a single treatment) manifest as rapid pain relief and improved cervical range of motion, with mechanisms involving activation of the descending pain inhibitory system and Aβ fiber-mediated “gate control” effects (18). The study by Gebrerufael et al. (59) indicated that patients showed significant pain reduction at 48-h follow-up after a single TSM session, but the effect size decreased over time. Long-term effects (after 2–4 weeks of treatment) require accumulation through multiple interventions, achieving sustained functional recovery through neuromuscular control reconstruction and central sensitization reversal. García-González et al. (60) in their 2024 RCT found that TSM combined with cervical manipulation resulted in disability index improvement exceeding the minimal clinically important difference (MCID) after one week, but changes in cervical range of motion did not reach significant thresholds, suggesting that long-term functional improvement requires multimodal intervention. Thus, research on the immediate effects of TSM in neck pain patients serves as the foundation for long-term effects and should not be overlooked—this is also one of the research objectives of the present study.

The randomized controlled trials analyzed in this study demonstrated that TSM therapy for cervical pain patients did not result in any documented severe adverse reactions. The incidence of side effects remained minimal, affecting only a small proportion of participants across all studies. Based on these findings and in accordance with the American Physical Therapy Association’s established protocols for TSM application as outlined in their clinical practice standards (61) this therapeutic approach appears to be a safe treatment option for individuals suffering from neck discomfort.

## Limitation

This meta-analysis presents certain constraints that warrant attention. Primarily, the majority of incorporated investigations featured limited participant numbers, which may distort treatment evaluations and inflate the perceived effectiveness of TSM. Additionally, substantial variation existed in the illness duration of enrolled participants across different randomized controlled trials, leading to notable discrepancies in short-term outcomes among neck pain patients receiving TSM therapy. Moreover, the selected RCTs predominantly focused on assessing TSM’s immediate therapeutic benefits for neck pain sufferers, with insufficient extended monitoring data to evaluate its sustained effects. Finally, due to the limited number of currently included studies with small sample sizes, we were unable to conduct subgroup analyses across more dimensions (population type, symptom duration, comparator interventions, dosage, technique standardization, and clinician experience). Additionally, detailed efficacy comparisons between different manipulation techniques could not be performed due to the limited variety of techniques employed. Similarly, because of the small number of included studies and the failure of many studies to adopt uniform types of assessment indicators, we were also unable to construct funnel plots; therefore, the stability of the study results could only be tested through sensitivity analysis using the “leave-one-out” method. Consequently, future research should prioritize large-scale, multicenter, double-blind randomized controlled trials with prolonged follow-up periods. Standardization of participant selection criteria based on disease duration would facilitate more accurate comparisons and analyses of TSM’s long-term effectiveness in managing neck pain, thereby offering more reliable clinical evidence. Substantial clinical evidence supports the therapeutic use of TSM in medical practice.

## Conclusion

In summary, TSM demonstrates rapid alleviation of discomfort and enhances cervical mobility across all movement planes for individuals suffering from neck discomfort. Nevertheless, the thrust-based approach within TSM yields superior outcomes compared to non-thrust mobilization methods. Furthermore, TSM exhibits an excellent safety record when administered to patients with neck pain, making it a viable option for widespread clinical adoption. For optimal therapeutic benefits, integrating thrust techniques with non-thrust mobilization strategies is advised, ensuring a more holistic approach to managing neck pain.

## Data Availability

The original contributions presented in the study are included in the article/Supplementary material, further inquiries can be directed to the corresponding author.
