# Supplementary material for: The immediate effects of thoracic spine manipulation in patients with neck pain: a meta-analysis of randomized controlled trials
Source: Front Med (Lausanne). 2026 Mar 31;13:1790614. doi: 10.3389/fmed.2026.1790614 (PMC13076109; doi:10.3389/fmed.2026.1790614)
Supplement: Supplementary file 1 [file Supplementary_file_1.docx]

**PubMed:**

#1 Neck pain[Mesh] OR Neck Pains[Title/Abstract] OR Pain, Neck[Title/Abstract] OR Pains, Neck[Title/Abstract] OR Neck Ache[Title/Abstract] OR Ache, Neck [Title/Abstract] OR Aches, Neck[Title/Abstract] OR Neck Aches[Title/Abstract] OR Cervicalgia[Title/Abstract] OR Cervicalgias[Title/Abstract] OR Cervicodynia[Title/Abstract] OR Cervicodynias[Title/Abstract] OR Neckache[Title/Abstract] OR Neckaches[Title/Abstract] OR Cervical Pain[Title/Abstract] ORCervical Pains[Title/Abstract] OR Pain, Cervical[Title/Abstract] OR Pains, Cervical[Title/Abstract] OR Posterior Cervical Pain[Title/Abstract] OR Cervical Pain, Posterior[Title/Abstract] OR Cervical Pains, Posterior[Title/Abstract] OR Pain, Posterior Cervical[Title/Abstract] OR Pains, Posterior Cervical[Title/Abstract] OR Posterior Cervical Pains[Title/Abstract] OR Posterior Neck Pain[Title/Abstract] OR Neck Pain, Posterior[Title/Abstract] OR Neck Pains, Posterior[Title/Abstract] OR Pain, Posterior Neck[Title/Abstract] OR Pains, Posterior Neck[Title/Abstract] OR Posterior Neck Pains[Title/Abstract] OR Anterior Cervical Pain[Title/Abstract] OR Anterior Cervical Pains[Title/Abstract] OR Cervical Pain, Anterior[Title/Abstract] OR Cervical Pains, Anterior[Title/Abstract] OR Pain, Anterior Cervical[Title/Abstract] OR Pains, Anterior Cervical[Title/Abstract] OR Anterior Neck Pain[Title/Abstract] OR Anterior Neck Pains[Title/Abstract] OR Neck Pain, Anterior[Title/Abstract] OR Neck Pains, Anterior[Title/Abstract] OR Pain, Anterior Neck[Title/Abstract] OR Pains, Anterior Neck[Title/Abstract]

#2 Cervical[Title/Abstract] OR Cervical Vertebrae[Title/Abstract] OR Vertebrae, Cervical[Title/Abstract] OR Cervical Spine[Title/Abstract] OR Cervical Spines[Title/Abstract] OR Spine, Cervical[Title/Abstract] OR C2 Vertebra[Title/Abstract] OR C2 Vertebras[Title/Abstract] OR

Epistropheus[Title/Abstract] OR Cervical Vertebra Axis[Title/Abstract] OR

Vertebra Axis, Cervical[Title/Abstract] OR Os Odontoideum[Title/Abstract]

#3 Cervical Spondylosis[Title/Abstract] OR Spondylosis, Cervical[Title/Abstract] OR Axial neck pain[Title/Abstract] OR Cervical Radiculopathy[Title/Abstract] OR Radiculopathy, Cervical[Title/Abstract] OR Cervical Radiculopathies[Title/Abstract] OR Radiculopathies, Cervical[Title/Abstract] OR Cervical myelopathy[Title/Abstract] OR Myelopathy, Cervical[Title/Abstract] OR Cervical disc disorders[Title/Abstract]

#4 #1 OR #2 OR #3

#5 Thoracic spine manipulation[Mesh] OR Thoracic spine manipulation[Title/Abstract] OR Thoracic vertebra Manipulation[Title/Abstract] OR Manipulation, Thoracic vertebra[Title/Abstract] OR Thoracic Manipulation[Title/Abstract] OR Manipulation, Thoracic[Title/Abstract] OR Orthopedic Manipulation[Mesh] OR Orthopedic Manipulation[Title/Abstract]

#6 chiropractic[Mesh] OR Manipulation, Orthopedic[Title/Abstract] OR Manipulation, Chiropractic[Title/Abstract] OR Manipulation, Chiropractic[Mesh] OR Chiropractic Manipulation[Title/Abstract] OR Spinal Adjustment, Chiropractic[Title/Abstract] OR Adjustment, Chiropractic Spinal[Title/Abstract] OR Adjustments, Chiropractic Spinal[Title/Abstract] OR Chiropractic Spinal Adjustment[Title/Abstract] OR Chiropractic Spinal Adjustments[Title/Abstract] OR Spinal Adjustments, Chiropractic[Title/Abstract] OR Chiropractic Adjustment[Title/Abstract] OR Adjustment, Chiropractic[Title/Abstract]

#7 Manipulation, Osteopathic[Mesh] OR Osteopathic Manual therapy[Title/Abstract] OR Osteopathic Manual therapys[Title/Abstract] OR Treatment, Osteopathic Manipulative[Title/Abstract] OR Treatments, Osteopathic Manipulative[Title/Abstract] OR Osteopathic Manipulation[Title/Abstract]

#8 #5 OR #6 OR #7

#9 (clinical[tiab] AND trial[tiab]) OR "clinical trials as topic"[mesh] OR "clinical trial"[pt] OR random*[tiab] OR "random allocation"[mesh] OR "therapeutic use"[sh]

#10 #4 AND #8 AND #9

**Cochrane Library:**

#1 MeSH descriptor: [Neck pain] explode all trees

#2 MeSH descriptor: [Cervicalgia] explode all trees

#3 MeSH descriptor: [Cervical Radiculopathy] explode all trees

#4 #1 OR #2 OR #3

#5 (TSM):ti,ab,kw

#6 (Thoracic spine manipulation):ti,ab,kw

#7 (Osteopathic Manual therapy):ti,ab,kw

#8 (Thoracic Spine Manipulative Therapy):ti,ab,kw

#9 (Thoracic vertebra Manipulation):ti,ab,kw

#10 (Thoracic Spinal Adjustments):ti,ab,kw

#11 #6 OR #7 OR #8 OR #9 OR #10

#12 #4 AND #11

**Web of Science:**

#1 TS=( Neck pain OR Neck Pains OR Pain, Neck OR Pains, Neck OR Neck Ache OR Ache, Neck OR Aches, Neck OR Neck Aches OR Cervicalgia OR Cervicalgias OR Cervicodynia OR Cervicodynias OR Neckache OR Neckaches OR Cervical Pain ORCervical Pains OR Pain, Cervical OR Pains, Cervical OR Posterior Cervical Pain OR Cervical Pain, Posterior OR Cervical Pains, Posterior OR Pain, Posterior Cervical OR Pains, Posterior Cervical OR Posterior Cervical Pains OR Posterior Neck Pain OR Neck Pain, Posterior OR Neck Pains, Posterior OR Pain, Posterior Neck OR Pains, Posterior Neck OR Posterior Neck Pains OR Anterior Cervical Pain OR Anterior Cervical Pains OR Cervical Pain, Anterior OR Cervical Pains, Anterior OR Pain, Anterior Cervical OR Pains, Anterior Cervical OR Anterior Neck Pain OR Anterior Neck Pains OR Neck Pain, Anterior OR Neck Pains, Anterior OR Pain, Anterior Neck OR Pains, Anterior Neck)

#2 TS= (Thoracic Spinal Manipulation OR Spinal Manipulation OR Thoracic Vertebra Manipulation OR Manipulation, Thoracic Vertebra OR Thoracic Manipulation OR Manipulation, Thoracic OR Orthopedic Manipulation OR Orthopedic Manipulation OR chiropractic OR Manipulation, Orthopedic OR Manipulation, Chiropractic OR Manipulation, Chiropractic OR Chiropractic Manipulation OR Spinal Adjustment, Chiropractic OR Adjustment, Chiropractic Spinal OR Adjustments, Chiropractic Spinal OR Chiropractic Spinal Adjustment OR Chiropractic Spinal Adjustments OR Spinal Adjustments, Chiropractic OR Chiropractic Adjustment OR Adjustment, Chiropractic OR Manipulation, Osteopathic OR Osteopathic Manual therapy OR Osteopathic Manual therapys OR Treatment, Osteopathic Manipulative OR Treatments, Osteopathic Manipulative OR Osteopathic Manipulation)

#3 TS= (clinical trial OR random* OR random allocation OR random* controlled trial OR placebo)

#4 #1 AND #2 AND #3

**Embase:**

#1 'neck pain'/exp OR 'cervicalgia'/exp OR 'cervical pain'/exp OR 'cervicalgia'/exp

#2 ('cervical vertebrae':ab,ti OR 'cervical spine':ab,ti OR 'cervical myelopathy':ab,ti OR 'cervical disc disorders':ab,ti) AND 'cervical radiculopathy':ab,ti

#3 #1 OR #2

#4 'smt':ti,ab OR ' Thoracic spinal manipulation therapy':ab,ti OR 'Thoracic vertebra Manual therapy':ti,ab OR ' Thoracic spinal manipulative therapy':ab,ti OR 'Thoracic vertebra manipulation':ab,ti OR 'Thoracic spinal adjustments':ab,ti

#5 'clinical':ti,ab AND 'trial':ti,ab OR 'clinical trial'/exp OR random*

#6 #3 AND #4 AND #5
